# Incidence and Contributing Factors of Persistent Hyperglycemia at 6–12 Weeks Postpartum in Iranian Women with Gestational Diabetes: Results from LAGA Cohort Study

**DOI:** 10.1155/2017/9786436

**Published:** 2017-04-12

**Authors:** Sedigheh Nouhjah, Hajieh Shahbazian, Nahid Shahbazian, Alireza Jahanshahi, Shayesteh Jahanfar, Bahman Cheraghian

**Affiliations:** ^1^Diabetes Research Center, Health Research Institute, Ahvaz Jundishapur University of Medical Sciences, Ahvaz, Iran; ^2^Department of Obstetrics and Gynecology, Fertility, Infertility and Perinatology Research Center, Imam Khomeini Hospital, Ahvaz Jundishapur University of Medical Sciences, Ahvaz, Iran; ^3^Internal Medicine Ward, Golestan Teaching Hospital, Ahvaz Jundishapur University of Medical Sciences, Ahvaz, Iran; ^4^School of Health Sciences, Central Michigan University, Building 2212, Room 2239, Mount Pleasant, MI 48859, USA; ^5^Department of Epidemiology and Biostatistics, Faculty of Health, Ahvaz Jundishapur University of Medical Sciences, Ahvaz, Iran

## Abstract

*Background*. A history of gestational diabetes is an important predictor of many metabolic disturbances later in life.* Method*. Life after gestational diabetes Ahvaz Study (LAGAs) is an ongoing population-based cohort study. Up to February 2016, 176 women with gestational diabetes underwent a 75 g oral glucose tolerance test (OGTT) at 6–12 weeks postpartum in Ahvaz (southwestern of Iran). Gestational diabetes was diagnosed according to the International Association of Diabetes and Pregnancy Study Groups (IADPSG) criteria and the American Diabetes Association (ADA) criteria applied for diagnosis of postpartum prediabetes and diabetes. Univariate and multivariate regression analysis were done.* Results*. Overall incidence of early postpartum glucose intolerance was 22.2% (95% CI, 16.3–29.0), 17.6% prediabetes (95% CI, 12.3–24.1) and 4.5% diabetes (95% CI, 2.0–8.8%). Independent risk factors for glucose intolerance were FPG ≥ 100 at the time of OGTT (OR 3.86; 95% CI; 1.60–9.32), earlier diagnosis of GDM (OR 0.92; 95% CI; 0.88–0.97), systolic blood pressure (OR 1.02; 95% CI; 1.002–1.04), and insulin or metformin therapy (OR 3.14; 95% CI; 1.20–8.21).* Conclusion*. Results determined a relatively high rate of glucose intolerance at 6–12 weeks after GDM pregnancy. Early postpartum screening of type 2 diabetes is needed particularly in women at high risk of type 2 diabetes.

## 1. Introduction

Gestational diabetes mellitus (GDM) is defined as any degree of glucose intolerance, onset or first recognition during pregnancy [1]. GDM is associated with adverse maternal and neonatal outcomes [2, 3]. The literature reports that GDM, through increased medical cost for prenatal care and excess cost of the newborn in the first year after birth, burdens a healthcare system. Based on a national report in the United States, GDM increased medical costs by $636 million ($596 million for maternal costs and $40 million for neonatal cost), although the long-term medical cost of GDM and related outcomes was not clarified [4].

Gestational diabetes mellitus, as a common metabolic disorder during pregnancy, affects between 1% and 28% of pregnancies [5] and global statistics indicate an even higher rate [6]. Incidence of GDM is increasing and this rise is attributed to obesity, type 2 diabetes and metabolic syndrome epidemic, advancing maternal age of pregnancy, and implementation of newly diagnosed criteria [1, 7, 8].

Prevalence of gestational diabetes in Iran varies from 1.3% to 18.6% based on Carpenter-Coustan criteria [9]. A Recent report shows high incidence of GDM in this area using new criteria (29.9%) [10].

A history of gestational diabetes is a strong predictor of many metabolic disturbances, particularly glucose intolerance later in life [11]. In the literature, the risk of diversion to type 2 diabetes increased during the 20-year period after the index pregnancy [12]. Reports have shown that the pattern can be changed and the interval shortened through changes in lifestyle [13, 14].

International guidelines recommend early screening of glucose status for development of prediabetes or diabetes in women with gestational diabetes at 6–12 weeks postpartum using the 75 g 2 h oral glucose tolerance test (OGTT) and thereafter [15–17]. But in most population, rates of women who return postpartum and screen are low [18, 19].

Potential predicators of future progression of prediabetes and/or diabetes in women with gestational diabetes are prepregnancy body mass index (BMI), higher blood glucose level in pregnancy, particular ethnic groups, earlier diagnosis of GDM, requirement of insulin therapy during pregnancy, and advanced maternal age, with high heterogeneity in reported risk factors [7, 20, 21].

Common universal features of GDM reported in recent decades include a rising trend in incidence of the condition [1] increasing prevalence of its risk factors [8, 11]; a high rate of metabolic and cardiovascular outcomes [11]; earlier progression to prediabetes and type 2 diabetes [13, 14], and suboptimal return of exposed women for postpartum screening and lifestyle interventions based on previous reports and our recent report in this area [18, 22] show this population is a high risk group for many metabolic outcomes. Therefore early identification of women with greater risk of progression to diabetes may provide opportunity for prevention and intervention program. Our motivation to conduct this study was lack of information on risk factors associated with early postpartum glucose intolerance following adoption of the International Association of Diabetes and Pregnancy Study Groups (IADPSG) criteria. we sought to estimate incidence of early postpartum glucose intolerance and to identify risk factors for future diabetes incidence among women with recent gestational diabetes.

## 2. Methods

### 2.1. Study Design and Settings

Life after gestational diabetes Ahvaz Study (LAGAs) is a population-based prospective cohort study to investigate potential short and long-term metabolic outcomes of gestational diabetes in mothers and their offspring. For this reason, the first gestational diabetes clinic in Khuzestan province was established as center of the cohort study on October 2013 in Golestan Hospital, the teaching hospital of Ahvaz Jundishapur University of Medical Sciences. Ahvaz is the capital of Khuzestan province, located in southwestern of Iran with high birth rate, high prevalence of obesity, metabolic syndrome, and type 2 diabetes [23–26].

### 2.2. Study Population

Pregnant women attending 25 urban public and private centers seeking prenatal care were recruited from March 2015. Single approach for screening, treatment, and management of GDM was applied. Initial screening was done in first trimester of pregnancy by fasting plasma glucose (FPG) test. Women with FPG ≥ 126 mg/dL and those who had pregestational diabetes type 1 or type 2 as defined by American Diabetes Association (ADA) criteria [27] were excluded. Women with FPG 100–125 mg/dL in first trimester of pregnancy received diet and physical activity program for 2 weeks and blood glucose check with 3 weeks interval. Based on results of blood test, women were referred for pharmacotherapy or continuing diet and physical activity. Women who refuse to continue and those planning to move from Ahvaz city within the subsequent 2 years were excluded.

75 g OGTT was performed between 24 and 32 weeks of gestation and based on IADPSG criteria, only one abnormal value equal to or greater than the known threshold value was detected as GDM values (≥92 for fasting, ≥180 and ≥153 mg/dL for 1-hour and 2-hour plasma glucose level, resp.) [28]. Women with impaired glucose metabolism, BMI ≥ 30 kg/m^2^, history of diabetes mellitus in first relatives, and history of GDM in previous pregnancies underwent earlier OGTT before 14th week of gestation [16] and screening was repeated between 24 and 32 weeks of gestation if the result was normal.

All women with gestational diabetes received dietary and exercise counseling, individualized diet or pharmacotherapy (insulin or metformin), and clinical and biochemical reassessment with 2-3 weeks' interval. ADA recommendations were used for goals of treatment [29].

A list of women who were diagnosed as GDM with details of their address and contact information were sent for research team and women referred to Golestan Hospital if they verbally accepted participation in the study. Various methods of communication were used to encourage women to participate and for retention including direct contact by phone, SMS texts via phone line, and other social networks (Whats app and telegram). Messages and reminders were sent to the women themselves and/or to husbands or other relatives who had made their contact number available. Communications were made by Sedigheh Nouhjah who was able to respond to any questions presented by the participants. Clinical evaluation and blood tests were free of charge and a copy of their laboratory reports was submitted to the patients for future reference.

### 2.3. Data Collection

Questionnaires related to sociodemographic characteristics of women and their husbands, potential risk factors of GDM, pregnancy outcome for the fetus and mothers, obstetrics and medical history, physical activity, and food habits of participants during 6–12 weeks postpartum were filled by a trained questionnaire. A comprehensive second questionnaire will be used as a follow-up within 2 years of the date of entering the study, based on predicted plane, to identify postpartum problems among mothers or their infants. The questionnaires were completed by an independent and trained researcher at the prenatal care center and postpartum data were collected by a member of the research team in our cohort center. Short forms of delivery were completed during 3–5 days after delivery in 2 main centers for screening of neonatal hypothyroidism.

### 2.4. Postpartum Biochemical and Clinical Assessment

FPG and 75 g 2-hour oral glucose tolerance test were performed at 6–12 weeks after delivery and these tests will be repeated annually for up to two years postpartum. Fasting plasma glucose ≥ 126 mg/dL or 2 hour plasma glucose after a 75 g oral glucose load ≥ 200 mg/dL was defined as diabetes, and prediabetes was diagnosed with fasting plasma glucose between 100 and 125 mg/dL or 2 h post-glucose loading level between 140 and 199 mg/dL according to ADA criteria [30]. Diagnosis of diabetes was confirmed if a second test was positive using the same criteria.

Blood samples were transported to the laboratory at the Diabetes Research Center, where the research coordinator and two laboratory technicians evaluated blood samples using the standard methods. Prepregnancy weight was recorded using medical and health profile. Weight, height, waist circumference, blood pressure, and hip circumference were measured at each visit during pregnancy and postpartum follow-up.

### 2.5. Statistical Analysis

Statistical analysis was performed with Statistical Package for the* Social* Sciences (SPSS version 22). Clinical, biochemical, and sociodemographic characteristics of women who progressed to postpartum glucose intolerance compared with women with normal glucose tolerance using univariate logistic regression analysis. Risk factors with *P* value less than 0.05 were tested in multivariate backward regression model.

### 2.6. Ethics Statement

The study protocol was approved by the Ethics Committee of Ahvaz Jundishapur University of Medical Sciences and all participants signed written informed consent form in addition to the first verbal agreement to taking part in the baseline visit.

## 3. Results

Until now, of 800 women who were diagnosed as GDM, 176 women (22%) aged between 17–45 years underwent a postpartum OGTT at 6–12 weeks after delivery. About half of these participants had high school education. Most of them were housewives (92.4%). The mean prepregnancy BMI was 27.82 kg/m^2^ (±4.41 SD). Records showing overweight or obesity were observed in 126/176 (71.6%) of participants based on prepregnancy BMI (Table 1). This rate was 79% (48.9% overweight and 30.1% obesity) using postpartum BMI. Birth weight ≥ 4000 gr was seen in 22 (12.5%) of newborns who had been exposed to GDM during the fetal period. A history of previous GDM was reported by 20.5% of multigravida participants.

Demographic and clinical characteristics associated with postpartum glucose test results are presented in Table 1. Mean gestational age at diagnosis of GDM was 19.83 weeks (range 4–37 weeks, ±8.46 SD). During oral glucose tolerance test, FPG ≥ 92 mg/dL, 1-hour plasma glucose level ≥ 180 mg/dL, and 2-hour plasma glucose concentration ≥153 mg/dL were detected in 65.9%, 34.7%, and 38.6% of women with gestational diabetes, respectively. Mean ± SD of clinical and laboratory characteristics of women with GDM based on results of postpartum 75 g OGTT are presented in Table 2.

The overall incidence of early postpartum glucose intolerance was 22.2% (95% CI, 16.3–29.0) inclusive of 17.6% prediabetes (95% CI, 12.3–24.1) and 4.5% diabetes (95% CI, 2.0–8.8%). Risk factors of persistent hyperglycemia at 6–12 weeks after GDM pregnancy were inclusive of FPG ≥ 100 at the time of OGTT in pregnancy, earlier diagnosis of GDM in pregnancy, systolic blood pressure, and the need for insulin inject or metformin therapy. Results of univariate and multivariate analysis using backward logistic regression are presented in Table 3.

## 4. Discussion

### 4.1. Postpartum Glucose Intolerance Rate

Incidence of glucose intolerance at 6–12 weeks after GDM pregnancy determined in this study was 22.5% (17.5% prediabetes and 4.5% type 2 diabetes).

These high estimated rates are consistent with results reported by Tovar et al. [31]. They reviewed 11 studies conducted between 2008 and 2010. Proportion of diabetes and prediabetes at 6–12 weeks postpartum were 1.2–4.5% and 12.2–36%, respectively. Rate of diabetes in our study was also consistent with that of Capula et al. [32] study on Caucasian women in Southern Italy at 6–12 weeks postpartum (4%) and Inturrisi et al. (4% to 9%) [33], but lower than previous reports in Asian women by Jang et al. (5–15%) [34, 35] and Korean women in Kwak et al. study (12.5%) [36].

In present study rate of prediabetes was in range with many previous reports [31] but lower than recent reports from Korea by Cho et al. (44.1%) [37] and that reported by Capula and colleagues (32.1%) [32].

Different ethnic groups with different backgrounds including prevalence of diabetes mellitus, obesity, metabolic syndrome, and variation in rate of severe hyperglycemia in pregnancy using different criteria for GDM diagnosis in pregnancy and classification of postpartum glucose intolerance may have caused this wide variation in rates.

### 4.2. Determinants of Postpartum Glucose Intolerance

Multivariate regression test showed that risk factors associated with persistent glucose intolerance 6–12 weeks postpartum were inclusive of earlier diagnosis of GDM, use of insulin or metformin for management of GDM, FPG ≥ 100 at the time of OGTT, and systolic blood pressure.

Various potential risk factors have been determined in other research for prediabetes or diabetes in early postpartum period previously [35, 38, 39]. Kwak et al. compared early and late clinical risk factors for progression of postpartum glucose intolerance. Except early detection of GDM and use of insulin, in early converters, no differences have been reported between the 2 groups (early and late* converters*) [36].

### 4.3. Gestational Age at Diagnosis

Consistent with results of this study, earlier diagnosis of GDM in pregnancy before increasing resistance to insulin between 16 and 26 weeks of gestation [7, 40] is reported as a common risk factor associated with progression to later glucose intolerance in women with GDM. Results of a systematic review on 39 studies including 95,750 women demonstrated higher risk of progress to abnormal glucose tolerance in women diagnosed with GDM in early weeks of gestation (RR 2.13, 95% CI 1.52–3.56) [20]. Although there is strong evidence in support of these results, some controversy remains over screening high risk pregnant women in earlier weeks of gestation [40].

### 4.4. Use of Insulin or Metformin

In the present study, pharmacotherapy (use of insulin or metformin) for management of hyperglycemia in pregnancy was strong predictor of progression to abnormal glucose tolerance at 6–12 weeks postpartum (OR 3.14; 95% CI; 1.20–8.21). Rate of postpartum glucose intolerance in women who were treated with insulin was 54.4% versus 41.7% in women who had taken metformin while the same rate was 15.4% in diet-only group.

Need for treatment with insulin or metformin as an indicator of severity of hyperglycemia may reflect impairment in *β*-cell function in women exposed to gestational diabetes. Pellonperä et al. studied women with GDM with two or more abnormal glucose values at 2 h 75 g OGTT, for a period of one year postpartum. This study showed higher incidence of prediabetes in metformin and insulin users (19.1% and 15.6%, resp.) than women treated with diet only (7.1%, *P* = 0.039). Also, the rate of diabetes was lower in the diet treatment group (*P* = 0.027) [41]. In addition, findings reported by Ziegler et al. [42] showed 92.3% of German women who used insulin in pregnancy progressed to diabetes during the 15-year period after GDM pregnancy versus 39.7% in the diet treatment group. Median years of diabetes-free duration in women who had taken insulin was 2.1 years. This duration in the diet-only treatment group was 16.3 years.

### 4.5. FPG during OGTT

Another risk factor for progression of abnormal glucose tolerance in present study was a higher level of FPG (≥100) at the time of OGTT, during a 24–32 weeks' gestation period (OR 3.86; 95% CI; 1.60–9.32).

Fasting glucose on OGTT is a common risk factor associated with prediabetes and diabetes after GDM pregnancy [14, 43, 44]. Moreover, elevated fasting glucose level at the time of OGTT had been a strong factor associated with insulin therapy in pregnancy. Different cut-off values based on applied criteria for diagnosis of GDM were reported for this association [45–47]. Another report by Langer showed that fasting glucose > 105 mg/dL was associated with fetal macrosomia, hypoglycemia in newborn infants, cesarean delivery, and a need for insulin therapy [48]. Previous studies have failed to determine an association between fasting glucose and future progression to type 2 diabetes for several reasons as follows: it was not measured [49]; the variable was not entered in the statistical model [50]; or modeling excluded GDM women with high fasting glucose level [39, 51] in the studies [7, 52].

### 4.6. Blood Pressure

This study determined systolic blood pressure as another related risk factor. Hypertensive disorder in pregnancy (RR 1.38; 95% CI; 1.32–1.45) was associated with future diabetes based on the systematic review and meta-analysis reported in Rayanagoudar et al. [20]. Feig et al. showed that women with hypertensive disorder in pregnancy had a 2-fold increased risk of developing diabetes by 16.5 years after pregnancy, even in women without GDM [53]. In addition, women with preeclampsia and gestational hypertension had insulin resistance independent of obesity and abnormal glucose tolerance during pregnancy [54, 55].

### 4.7. Other Factors

In the present study there was no association of maternal age at pregnancy, maternal education and occupation, parity, and family history of diabetes with development of glucose intolerance in the early postpartum period. Some previous studies have shown an association of maternal age, parity, and a family history of diabetes with glucose intolerance, but reports are inconsistent [7, 39, 52].

No significant association was determined between total weight gain during pregnancy, BMI, and obesity before pregnancy and postpartum glucose intolerance at 6–12 weeks postpartum. Women with higher blood glucose levels, who had a high chance of early postpartum progression to abnormal glucose intolerance, usually received a strict diet program that controlled their weight gain during pregnancy [56]. Body mass index was also reported as a significant risk factor for progression to glucose intolerance after GDM pregnancy in previous studies, but there was no consistency after adjusting for other covariates. The studies that show this association resulted from long-term evaluation of a GDM group [57, 58]. Similar to results of this study, other research has reported no association between BMI and glucose intolerance in early postpartum follow-up [7].

In present study 56.8% of women with gestational diabetes underwent cesarean delivery. High rate of cesarean delivery (48%) in Iranian population is reported by Azami-Aghdash et al. [59]. Mean birth weight was 3378 gr (±557 gr SD) in GDM-exposed newborns. Results of a systematic review conducted by Mirmiran et al. showed mean weight of Iranian newborns at birth has been 3152 gr (95% CI = 3114–3189) [60].

Moreover, based on our results, rate of preterm delivery was 8%. This rate in general population reported 9.2% (95% CI: 7.6–10.7) by Vakilian et al. [61], based on the random effect model in a systematic meta-analysis.

Preterm delivery was associated with higher risk of progression to postpartum glucose intolerance using univariate analysis but was not determined as a risk factor when adjusted in the multivariate model. Rayanagoudar et al. showed preterm delivery increases risk of diabetes in women with GDM (RR 1.81; 95% CI; 1.35–2.43) [20]. Our results related to preterm delivery could probably reach a significant level with increased number of subjects during future months of the study. Also, the role of some variables may change in a long-term follow-up.

### 4.8. Limitation and Strength

There were two main limitations in this study. Despite using different methods of communication, the rate of postpartum return for glucose testing and follow-up was low. This may be a source of bias. Participants in this study were compared in terms of sociodemographic and clinical characteristics and those who did not return for follow-up. There were no significant differences between the two groups. A similar problem has been reported in several previous studies [18, 19]. Another limitation was the relative small sample size. Ongoing sampling in future months may change the chances of some variables as significant risk factors and improve a wide range of confidence interval (CI) in multivariate analysis.

The strength of this study was that it involved husbands of patients at follow-up in order to decrease the chance of attrition bias. In addition, using a combination of different methods for recall and by sending reminders, this study benefited from its use of social media. To the best our knowledge, this study is the first population-based prospective cohort study in this area to follow postpartum metabolic outcome of gestational diabetes in mothers and their offspring that included several variables.

## 5. Conclusion

Gestational diabetes is a risk factor for postpartum progression to glucose intolerance. Short-term postpartum follow-up of women with GDM showed a relatively high rate of glucose intolerance among women in this population. This finding highlighted the need for early postpartum screening and implementation of an intervention program to prevent type 2 diabetes, particularly in women whose GDM was diagnosed during the earlier weeks of gestation, mothers with higher fasting glucose level at the time of OGTT, higher blood pressure in pregnancy, and patients who required pharmacotherapy for management of hyperglycemia.

## Figures and Tables

**Table 1:** Demographic and clinical characteristics associated with postpartum glucose test results among women with recent gestational diabetes at 6–12 weeks postpartum.

Characteristics	Total (*N* = 176)	NGT (*N* = 137)	AGT (*N* = 39)	*P* value^*∗*^
	*N* (%)	(%)	*N* (%)	
*Education*				
<high school	54 (30.7)	47 (34.3)	7 (17.9)	0.03
High school	81 (46.0)	56 (40.9)	25 (64.1)	
Collage	41 (23.3)	34 (24.8)	7 (17.9)	

*Gravidity*				
1	49 (27.8)	38 (27.7)	11 (28.2)	0.58
2	57 (32.4)	42 (30.7)	15 (38.5)	
3	40 (22.7)	31 (22.6)	9 (23.1)	
≥4	30 (17.0)	26 (19.0)	4 (10.3)	

*Ethnicity*				
Fars	63 (35.8)	47 (34.3)	16 (41.0)	0.29
Lor	33 ( 18.8)	29 (21.2)	4 (10.3)	
Arab	80 (45.5)	61 (44.5)	19 (48.7)	

*Prepregnancy BMI* ^*∗∗*^				
Normal	50 (28.4)	43 (31.4)	7 (17.9)	0.20
Overweight	73 (41.5)	56 (40.9)	17 (43.6)	
Obesity	53 (30.1)	38 (27.7)	15 (38.5)	

*Preterm delivery (<37 weeks' gestation)*				
Yes	14 (8.0)	7 (5.1)	7 (17.9)	**0.009**
No	162 (92.0)	130 (94.9)	32 (82.1)	

*Mode of delivery*				
Vaginal	76 (43.2)	64 (46.7)	12 (30.8)	0.07
Cesarean section	100 (56.8)	73 (53.3)	27 (69.2)	

*History of GDM in family*				
Yes	64^*∗∗∗*^(46.7)	47 (42.7)	17 (63.0)	0.05
No	73 (53.3)	63 (57.3)	10 (37.0)	

*History of diabetes in family*				
Yes	98 (55.7)	76 (55.5)	22 (56.4)	0.57
No	78 (44.3)	61 (44.5)	17 (43.6)	

*Hypertension in pregnancy*				
Yes	26 (14.8)	17 (12.4)	9 (23.1)	0.09
No	150 (85.2)	120 (87.6)	30 (76.9)	

*Nutrition of infant 6–12 weeks after birth*				
Breast feeding	131 (74.4)	102 (74.5)	29 (74.4)	0.99
Formula or combined	45 (25.6)	35 (25.5)	10 (25.6)	

*Treatment of GDM*				
Nutrition	142 (80.7)	120 (87.6)	22 (56.4)	<**0.001**
Metformin	12 ( 6.8)	7 (5.1)	5 (12.8)	
Insulin	22 (12.5)	10 (7.3)	12 (30.8)	

*Number of abnormal tests in pregnancy*				
1	87 (49.4)	71 (51.8)	16 (41)	0.40
2	41 (23.3)	31 (22.6)	10 (25.6)	
3	27 (15.3)	18 (13.1)	9 (23.1)	
≥4	21 (11.9)	17 (12.4)	4 (10.3)	

NGT, normal glucose tolerance; AGT, abnormal glucose tolerance (prediabetes or diabetes); GDM, gestational diabetes mellitus.

^*∗*^Using chi square test.

^*∗∗*^Normal ≤ 29.9 kg/m^2^, overweight ≥ 25kg/m^2^, obesity ≥ 30 kg/m^2^.

^*∗∗∗*^Missing data are unknown history of GDM in family.

**Table 2:** Clinical and laboratory characteristics of women with GDM based on results of 6–12 weeks postpartum glucose tolerance using 75-g OGTT.

Characteristics	Women with GDM (*N* = 176)	Normal glucose tolerance (*N* = 137)	Abnormal glucose tolerance (*N* = 39)	*P* value^*∗*^
	Mean (±SD)	Mean (±SD)	Mean (±SD)	
Age (years)	29.68 (5.25)	29.71 (5.50)	29.56 (4.29)	0.87
Gestational age of diagnosis	19.83 (8.46)	21.29 (8.12)	14.71 (7.86)	**<0.001**
Total weight gain in pregnancy (kg)	10.88 (6.29)	10.85 (6.67)	10.98 (4.78)	0.90
Weight before pregnancy (kg)	71.51 (12.10)	71.08 (11.67)	73.05 (13.57)	0.37
Weight in last month of pregnancy (kg)	82.33 (12.92)	81.83 (12.51)	84.06 (14.32)	0.34
Gestational age at birth (weeks)	38.97 (1.36)	39.05 (1.29)	38.66 (1.54)	0.11
Birth weight of the child (g)	3378.70 (557.33)	3450.28 (549.61)	3250.21 (572.36)	0.10
Systolic BP pregnancy (mmHg)	114.11 (20.26)	111.89 (16.32)	121.92 (124.05)	**0.003**
Diastolic BP (mmHg)	69.28 (12.38)	68.10 (11.14)	73.46 (15.43)	**0.01**
FPG first visit pregnancy (g/dL)	92.41 (11.63)	91.15 (10.17)	96.84 (15.06)	**0.007**
FPG at time of OGTT (g/dL)	97.80 (19.44)	95.06 (17.23)	107.41 (23.57)	<0.001
1-hour pregnancy (g/dL)	160.75 (35.80)	158.69 (36.92)	167.97 (30.94)	0.15
2-hour pregnancy (g/dL)	139.74 (35.72)	136.21 (35.73)	152.12 (33.26)	**0.01**
FPG postpartum (g/dL)	91.81 (18.25)	85.78 (7.52)	112.97 (27.24)	**<0.001**
2-hour 75 gr OGTT postpartum (g/dL)	106.19 (41.70)	93.99 (19.09)	149.05 (65.70)	**<0.001**
Postpartum BMI (kg/m^2^)	28.30 (4.43)	27.86 (4.21)	29.86 (4.86)	**0.01**

^*∗*^Using *t*-test.

GDM, gestational diabetes mellitus; BP, blood pressure; FPG, fasting plasma glucose; OGTT, oral glucose tolerance test; BMI, body mass index, defined as weight (kg)/height (m)^2^.

**Table 3:** Independent risk factors for glucose intolerance (prediabetes or diabetes) early postpartum using univariate and multivariate logistic regression analysis.

Variables	Unadjusted ORs	CI	*P* value	Adjusted ORs^*∗*^	95% CI	*P* value
Gestational age at diagnosis	0.90	0.86–0.95	<0.001	0.92	0.88–0.97	0.006
FPG of first visit pregnancy	1.04	1.01–1.07	0.008	1.04	0.93–1.02	0.27
FPG on OGTT pregnancy ≥ 100	6.10	2.83–13.4	<0.001	3.86	1.60–9.32	0.003
2-hour plasma glucose pregnancy	1.01	1.002–1.022	0.016	1.005	0.99–1.01	0.45
BP systolic pregnancy	1.02	1.007–1.04	0.006	1.02	1.002–1.04	0.032
Insulin or metformin treatment	5.45	2.42–12.28	<0.001	3.14	1.20–8.21	0.020
Postpartum BMI	1.10	1.01–1.19	0.015	1.04	0.94–1.15	0. 39
Preterm delivery (<37 weeks gestation)	4.06	1.33–12.41	0.014	3.17	0.83–12.4	0.091

^*∗*^The model is adjusted for covariates which had statistical *P* value less than 0.05 in the univariate analysis.
